# Melatonin Alleviates Neonatal Necrotizing Enterocolitis by Repressing the Activation of the NLRP3 Inflammasome

**DOI:** 10.1155/2022/6920577

**Published:** 2022-03-17

**Authors:** Xiaoyu Xiong, Zhongkun Bao, Yanhong Mi, Xinhong Wang, Jiajun Zhu

**Affiliations:** ^1^Department of Neonatology, Women's Hospital, Zhejiang University School of Medicine, China; ^2^Department of Radiology, Women's Hospital, Zhejiang University School of Medicine, China; ^3^Department of Radiology, The Second Affiliated Hospital, Zhejiang University School of Medicine, China

## Abstract

**Objective:**

Necrotizing enterocolitis (NEC) is one of the commonest gastrointestinal critical diseases in newborns. Several researches have proven the efficacy of melatonin (MEL) on NEC, but the latent mechanisms were ambiguous. We designed the current research to evaluate the function and mechanism of MEL on NEC in a neonatal mouse model.

**Methods:**

The newborn mice were subjected to formula milk containing LPS and hypoxia to establish a NEC model and also intraperitoneally injected with MEL. During the experiment, all mice were closely monitored and weighed. The effect of MEL on the histopathological injury of the terminal ileum tissues, inflammation, and oxidative stress of serum in NEC mice was examined by hematoxylin-eosin (H&E) staining and ELISA. The effect of MEL on the NOD-like receptor family pyrin domain containing 3 (NLRP3) inflammasome was assessed via quantitative real-time PCR and Western blot.

**Results:**

MEL intensified the survival rate and body weight in NEC mice. The H&E staining illustrated that MEL improved the histopathological injury in NEC mice. Moreover, MEL repressed the IL-1*β*, TNF-*α*, and MDA levels of serum and enhanced the SOD and GSH-Px levels of serum in NEC mice. We also discovered that MEL attenuated the mRNA and protein levels of NLRP3, Toll-like Receptor 4 (TLR4), NF-*κ*B, and caspase-1 of the terminal ileum tissues in NEC mice.

**Conclusion:**

Our research illuminated that MEL attenuated the severity of NEC via weakening the activation of the NLRP3 inflammasome.

## 1. Introduction

Necrotizing enterocolitis (NEC) is a serious gastrointestinal disease that occurs in neonates [1]. NEC often leads to systemic sepsis, multiple organ failure, and even death, which seriously threatens the health of neonates. NEC is characterized by intestinal injury (from mucosal injury to full-thickness necrosis and perforation), and its clinical manifestations are mainly feeding intolerance, shock, abdominal distension, and intestinal bleeding [2, 3]. It was proven that more than 90% of NEC patients are preterm infants, especially very low birth weight babies (weight < 1500 g) [4]. Since the discovery of NEC, many advances have been made in etiology, diagnosis, and treatment, but there are also some mechanisms that have not yet been elucidated [5]. Due to the unclear pathogenesis, the current prevention and treatment methods of NEC are also limited, mainly including breastfeeding, probiotics, antibiotics, and surgery [6, 7]. However, antibiotics cannot be used for a long time [8]; surgery can also cause a series of complications, such as sepsis, peritonitis, and wound infection, and the postoperative mortality and reoperation rates are both high (postoperative mortality is about 40%-50%, and the complication rate is about 40%-70%) [9]. Therefore, finding new and effective therapeutic targets and drugs is the focus of current research.

Mounting evidence manifested that inflammatory response generates a critical function in the progression of NEC [10, 11]. NOD-like receptor family pyrin domain-containing 3 (NLRP3), as a protein complex, is composed of upstream NLRP3, apoptosis-associated speck like protein (ASC), and caspase-1 [12]. The NLRP3 inflammasome responds to multiple signals. The activation of the NF-*κ*B pathway upregulates the expressions of NLRP3 and pro-IL-1*β*, while the activated NLRP3 inflammasome promotes neutrophil recruitment, leading to acute inflammatory responses [13]. The results exhibited that NLRP3 is involved in the inflammatory response during the development of NEC, so the effective inhibition of the activation of the NLRP3 inflammasome may provide insights for the development of new drugs for the therapy of NEC [14].

Melatonin (MEL), a neuroendocrine hormone secreted by the pineal gland, is a derivative of 5-hydroxytryptophan [15]. MEL has a variety of physiological functions, including anti-inflammatory, antioxidant, immune regulation, biological rhythm modulation, and tumor prevention [16, 17]. It was proven that MEL can inhibit the inflammatory response by weakening NLRP3 inflammasome activation in the process of atherosclerosis, acute lung injury, and hypoxic-ischemic brain injury [18–20]. Scientific research proved that MEL alleviated NEC via preventing Th17/Treg imbalance by AMPK/SIRT1 signaling activation [21]. Guven et al. illustrated that MEL notably attenuated the severity of NEC in a neonatal rat model [22]. However, whether MEL ameliorates NEC intestinal inflammatory damage by restraining the inflammatory response mediated by the NLRP3 inflammasome remains fuzzy.

Based on the above research evidence, this study constructed a neonatal NEC mouse model to probe the influence of MEL on intestinal inflammatory injury of neonatal NEC and its effect on the activation of the NLRP3 inflammasome in intestinal tissues, in order to provide new ideas and theoretical foundation for the therapy of neonatal NEC.

## 2. Materials and Methods

### 2.1. Ethics Statement

The animal experiment protocols were performed based on the guidelines of the China Animal Care and Use Committee. The research was ratified by Hangzhou Eyong Biotechnological Co., Ltd. Animal Experiment Center (Certificate No. SYXK (Zhe)2020-0024). All efforts were made to mitigate the agony of animals.

### 2.2. Experimental Animal

A total of 36 ten-day-old C57BL/6 mice with SPF grade were acquired from Shanghai Sippr-BK Laboratory Animal Co., Ltd. (Certificate No. SCXK (Hu)2018-0006; Shanghai, China). All mice were raised in a clean facility (22-24°C, relative humidity of 40–60%, under a 12 h day-night cycle) with free access to standard rodent chow and water.

### 2.3. NEC Model Establishment and Grouping

The mice were distributed into the control group (control), model group (NEC), and NEC+MEL group, with 12 mice in each group. The mice in the control group were fed in the same cage as the mother mice without other treatments. The mice in the NEC group were subjected to formula milk and hypoxia to establish a NEC model [21]. Briefly, mice were given formula milk (Similac Advance Infant Formula: Puppy-Canine Milk replacement, 2 : 1) for 4 days, and 4 *μ*g/g/day LPS was placed into the infant formula on days 2 and 3. Simultaneously, the mice were placed in a modular chamber containing 5% O_2_ and 95% N_2_ for 10 min, twice a day for 4 days. Each mouse in the NEC+MEL group was intraperitoneally injected with 100 *μ*L of MEL (M5250, Sigma-Aldrich, USA) at a dose of 10 mg/kg per day before the NEC module processing until the end of the experiment. The MEL in this study was dissolved in 0.9% NaCl and immediately administered to mice. Mice in the other groups were subjected to 0.9% NaCl in the same way. All mice were closely monitored and weighed at days 10, 12, and 14 after birth.

### 2.4. Tissue and Blood Sample Collection

Before execution, we took blood samples from the mouse's orbit. All samples were centrifuged at 1000 g for 20 min [23], and then, serum was obtained. All serum samples are stored at -80°C. On the 5th day after NEC induction (day 15 after birth), all mice were euthanized by cervical dislocation. After the abdominal incision, we removed the terminal ileum tissue from the mice. Part of the tissues was stored at -80°C for qRT-PCR and Western blot analysis. The remaining tissues were fixed with 4% paraformaldehyde, which were then embedded in paraffin. They were cut into 5 *μ*m sections for H&E staining.

### 2.5. H&E Staining

The ileum tissue slices were subjected to dewaxing with xylol, which were then rehydrated with ethanol gradient. After rinsing, we utilized hematoxylin (G1004, Servicebio, China) to stain the slices. After differentiating, the slices were subjected to stain with 1% eosin solution (C0109, Beyotime, China). Then, the results were observed using an optical microscope (Z723975-1EA, Sigma, USA) after dehydrating and sealing. The histopathological score was estimated by two independent pathologists who were unaware of the study conditions based on the histopathological scoring system described by Gao et al. [24]. Grade 0 represents normal intestine; grade 1 represents separation of the Villous core; grade 2 shows epithelial cell abscission to the level of the middle villi; grade 3 indicates necrosis of the entire villi. Tissues with a histological score of ≥2 are considered to manifest NEC.

### 2.6. ELISA

Mouse TNF-*α* Kit (PT512) and mouse IL-1*β* Kit (PI301) were obtained from Beyotime (China). The prepared serum samples were added to the sample well and placed at room temperature for 120 min. After washing, each well was reacted with mouse IL-1*β* biotinylated antibody/TNF-*α* biotinylated antibody at 37°C for 1 h followed by reaction with HRP-labeled streptavidin at 37°C in the dark for 20 min. After reacting with TMB solution at 37°C away from light for 20 min, each well was then subjected to treatment with stop solution. Lastly, we utilized a microplate reader (CMaxPlus, MD, USA) to measure the absorbance (450 nm).

### 2.7. Detection of MDA, SOD, and GSH-Px

MDA kit (A003-1-2), SOD kit (A001-3-2), and GSH-Px kit (A005-1-2) were acquired from Jiancheng (China). The contents of MDA, SOD, and GSH-Px in the serum samples were assessed by the corresponding kit based on the manufacturer's instruction.

### 2.8. Quantitative Real-Time PCR (qRT-PCR)

We utilized Total RNA Extractor (R1200, Solarbio, China) to extract RNA from the terminal ileum tissues. Subsequently, qRT-PCR reaction was carried out by using BeyoFast™ Probe One-Step qRT-PCR Kit (D7277M, Beyotime, China) under a PCR system (EDC-810, Eastwin Life Sciences, Inc.). GAPDH was taken as the normalization control. The primers were listed 5′ to 3′: NLRP3, (F) ATTACCCGCCCGAGAAAGG; (R) TCGCAGCAAAGATCCACACAG; TLR4, (F) AACCTGCTCTACCTACACCTG; (R) CCGAGAGATTGAGGAATCGAAG; NF-*κ*B, (F) AAGATCCGTATGGCCCTCAAA; (R) TCCATCCTGGATACTGAAAGAGT; caspase-1, (F) TACTCCCACCGTTGAGCTGT; (R) CCGTAGCATCTGTGGATAGGC; and GAPDH, (F) TGACCTCAACTACATGGTCTACA; (R) CTTCCCATTCTCGGCCTTG. These gene expressions were counted by the 2^−ΔΔCT^ method.

### 2.9. Western Blot

The harvested terminal ileum tissues were lysed via RIPA buffer (R0010, Solarbio, China). Next, we utilized the BCA kit (pc0020, Solarbio, China) to measure the concentrations of protein. The proteins were subjected to protein electrophoresis. Thereafter, the protein in the gel was shifted to a nitrocellulose membrane. After that, we utilized 5% nonfat milk to seal the membrane. The sealed membrane was washed and then exposed to primary antibodies all night at 4°C. The next day, the bound antibodies reacted with the anti-rabbit secondary antibody for 1.5 h at 37°C. After visualizing with the ECL reagent (abs920, Absin, China), the blots were placed in a ChemiScope 3300 Mini equipment (Clinx, China). The primary antibodies of anti-NLRP3 antibody (DF7438), anti-TLR4 antibody (AF7017), anti-NF-*κ*B antibody (AF5006), anti-caspase-1 antibody (AF5418), and anti-GAPDH antibody (AF7021) were acquired from Affinity (USA).

### 2.10. Statistical Analysis

SPSS software (16.0, IBM, USA) was utilized to process data. Measurement data between multiple groups were tested for normal distribution using the Shapiro-Wilk test. One-way ANOVA analysis was employed for comparing the differences among multiple groups. Comparison between groups was assessed by the SNK test. The Kruskal-Wallis *H* test was utilized to those with uneven variance. Data were expressed as mean ± standard deviation. The statistical significance was defined as *P* < 0.05.

## 3. Results

### 3.1. MEL Intensified the Survival Rate and Body Weight of NEC Mice

**Figure 1:**
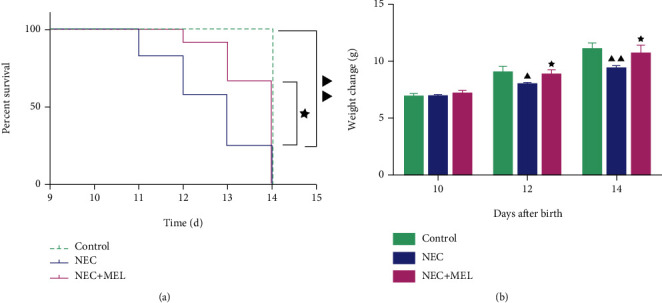
MEL intensified the survival rate and body weight in NEC mice. (a) MEL enhanced the survival rate in NEC mice. (b) MEL elevated body weight in NEC mice. ^▲^*P* < 0.05, ^▲▲^*P* < 0.01 vs. control; ^★^*P* < 0.05 vs. NEC. NEC: necrotizing enterocolitis; MEL: melatonin.

We first established a NEC mouse model and monitored the death of mice during the experiment. As exhibited in Figure 1(a), no mice in the control group died during the experiment. The mice in the NEC group died on day 11, and the survival rate on day 14 was less than 50% (Figure 1(a), *P* < 0.01). While after MEL intervention, the mortality of mice was greatly decreased, and the survival rate on day 14 was higher than 50% (Figure 1(a), *P* < 0.05). The body weight of mice in the NEC group was lower than that of the control group on day 12 and day 14 after birth (Figure 1(b), *P* < 0.05). More importantly, MEL notably improved the body weight of NEC mice born on day 12 and day 14 (Figure 1(b), *P* < 0.05).

### 3.2. MEL Improved the Histopathological Injury in NEC Mice

**Figure 2:**
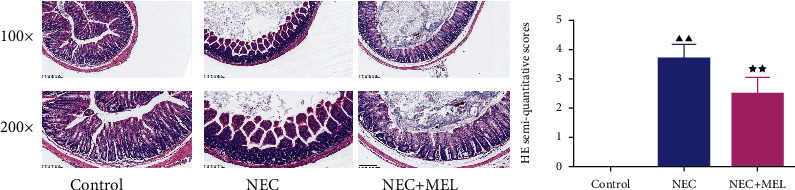
MEL improved the histopathological injury in NEC mice. The effect of MEL on the histopathological injury in NEC mice was examined by H&E staining. The histopathological score was estimated by two independent pathologists who were unaware of the study conditions based on the histopathological scoring system. ^▲▲^*P* < 0.01 vs. control; ^★★^*P* < 0.01 vs. NEC.

In the next research, we utilized H&E staining to assess the histopathological injury in NEC mice. As displayed in Figure 2, in the control group, the intestinal tissue structure was clear and the intestinal epithelium was neatly arranged. In the NEC group, the integrity of the intestinal tissue was injured, the intestinal epithelium was broken and some of it was necrotic and exfoliated or disappeared, and the muscle layer became thinner or even broken (Figure 2). However, the intestinal tissue structure in the NEC+MEL group was obviously improved compared with that in the NEC group (Figure 2). Moreover, the semiquantitative score of H&E in the NEC group was higher than that in the control group (Figure 2). The H&E semiquantitative score of the NEC+MEL group was extremely lower than that of the NEC group (Figure 2). It was unveiled that MEL ameliorated the histopathological injury in NEC mice.

### 3.3. MEL Weakened the Inflammation and Oxidative Stress of Serum in NEC Mice

**Figure 3:**
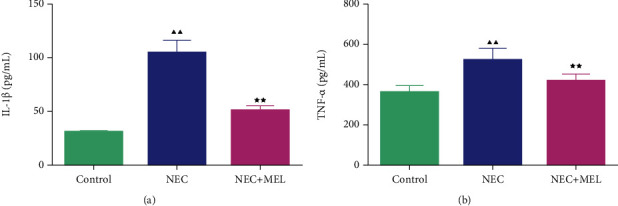
MEL weakened inflammation of serum in NEC mice. (a, b) The effect of MEL on inflammation in NEC mice was assessed by ELISA detecting the serum interleukin 1*β* (IL-1*β*) and tumor necrosis factor-alpha (TNF-*α*). ^▲▲^*P* < 0.01 vs. control; ^★★^*P* < 0.01 vs. NEC.

**Figure 4:**
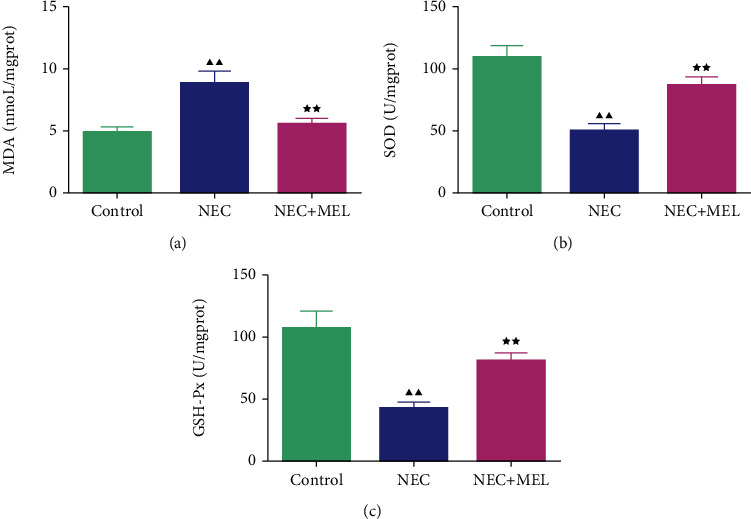
MEL weakened oxidative stress of serum in NEC mice. (a–c) The effect of MEL on oxidative stress in NEC mice was estimated by using a kit examining the serum malondialdehyde, superoxide dismutase, and glutathione peroxidase. ^▲▲^*P* < 0.01 vs. control; ^★★^*P* < 0.01 vs. NEC.

To check the role of MEL on inflammation- and oxidative stress-related markers of serum in NEC mice, we examined the contents of IL-1*β*, TNF-*α*, MDA, SOD, and GSH-Px. We discovered that the contents of IL-1*β* and TNF-*α* in NEC mice were largely augmented compared to the control mice, whereas MEL dramatically weakened the contents of IL-1*β* and TNF-*α* in NEC mice (Figures 3(a) and 3(b), *P* < 0.05). Moreover, the contents of SOD and GSH-Px were attenuated and the content of MDA was intensified in NEC mice (Figures 4(a)–4(c), *P* < 0.01). MEL notably boosted the contents of SOD and GSH-Px while it restrained the content of MDA in NEC mice (Figures 4(a)–4(c), *P* < 0.01).

### 3.4. MEL Attenuated the NLRP3, TLR4, NF-*κ*B, and Caspase-1 of the Terminal Ileum Tissues in NEC Mice

**Figure 5:**
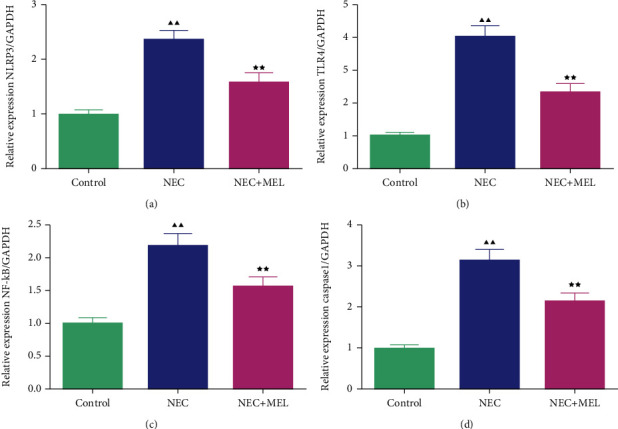
MEL attenuated the mRNA levels of NLRP3, TLR4, NF-*κ*B, and caspase-1 of the terminal ileum tissues in NEC mice. (a–d) The effect of MEL on the mRNA levels of NOD-like receptor family pyrin domain containing 3 (NLRP3), Toll-like Receptor 4 (TLR4), NF-kappaB (NF-*κ*B), and caspase-1 of the terminal ileum tissues in NEC mice was estimated by quantitative real-time PCR (qRT-PCR). ^▲▲^*P* < 0.01 vs. control; ^★★^*P* < 0.01 vs. NEC.

**Figure 6:**
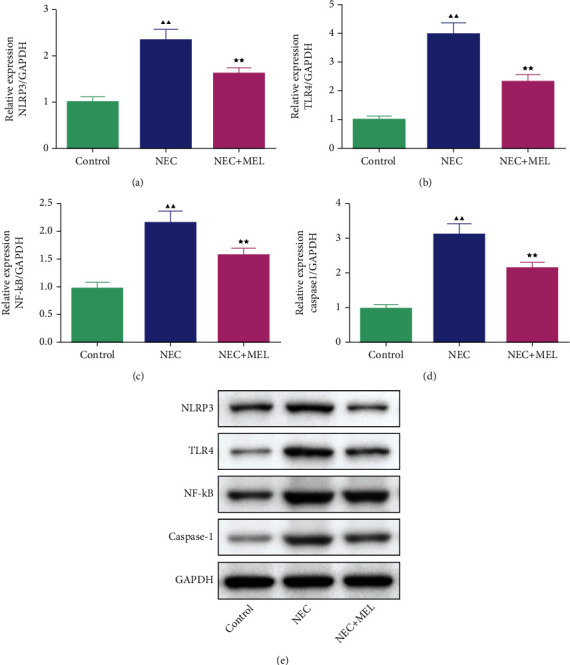
MEL attenuated the protein levels of NLRP3, TLR4, NF-*κ*B, and caspase-1 of the terminal ileum tissues in NEC mice. (a–d) The effect of MEL on the protein levels of NLRP3, TLR4, NF-*κ*B, and caspase-1 of the terminal ileum tissues in NEC mice was estimated by Western blot. ^▲▲^*P* < 0.01 vs. control; ^★★^*P* < 0.01 vs. NEC.

The inflammatory TLR4/NLRP3 pathway-related markers were further detected by qRT-PCR and Western blot. The qRT-PCR assay manifested that the mRNA levels of NLRP3, TLR4, NF-*κ*B, and caspase-1 of the terminal ileum tissues in NEC mice were prominently upregulated, while the elevated effects were partially offset by MEL treatment (Figures 5(a)–5(d), *P* < 0.01). We further utilized Western blot to examine the TLR4/NLRP3 pathway-related factors. The results of Western blot were consistent with qRT-PCR (Figures 6(a)–6(e), *P* < 0.01).

## 4. Discussion

In this work, the experimental NEC model was established in ten-day-old C57BL/6 mice by formula milk with LPS and hypoxia [21]. H&E staining results clarified that in the NEC group, the integrity of the intestinal tissue was lost, the intestinal epithelium was broken and some of it was necrotic and exfoliated or disappeared, and the muscle layer became thinner or even broken, illuminating that the mouse model of NEC was established successfully. More importantly, MEL improved the histopathological injury in NEC mice.

At present, epidemiology suggests that NEC is considered to be related to factors such as premature birth, intestinal mucosal hypoxia-ischemia, infection, and formula feeding [25]. The incidence of NEC in formula-fed newborns was notably higher than that in breast-fed newborns [26]. A study discovered that formula feeding within 10 days after birth evidently boosted the risk of NEC in infants, while breastfeeding did the opposite [27]. These studies demonstrated that formula facilitated the development of NEC, while breast milk has a certain preventive effect on NEC. In our study, we observed that there was no death in the breast-fed control mice, while NEC model rats had an obvious increase in mortality and a notable decrease in body weight after modeling. Interestingly, MEL intensified the survival rate and body weight in NEC mice, which was consistent with the results of Guven et al. [22].

Although it is not yet distinct which factors play a leading role in NEC, the theory that inflammatory factors participate in intestinal injury of NEC has gradually been recognized [28]. The proinflammatory pathway mediated by TLR4 is a crucial link in the pathogenesis of neonatal NEC, and inhibition of the TLR4-mediated inflammatory pathway may be a new therapy for the prevention and therapy of neonatal NEC [29, 30]. The key link between TLR4 overexpression and apparent inflammatory disease may be the production of downstream IL-1*β* [31]. IL-1*β* can induce intestinal inflammation and generate a modulation in epithelial healing and repair by recruiting and activating immune cells to further induce the generation of chemokines, proinflammatory cytokines, and growth factors [32]. The maturation and secretion of IL-1*β* are highly controlled by the activation of NLRP3 induced by TLR4/NF-*κ*B to prevent inappropriate inflammation [33, 34]. The NLRP3 inflammasome could activate caspase-1, mediate the maturation and secretion of IL-1*β* and IL-18, and further recruit and activate other immune cells to enhance local and systemic inflammatory responses [35]. In addition, it was confirmed that TNF-*α* induces intestinal mucosal damage by activating the inflammatory cascade, advancing the release of itself and other inflammatory factors [36].

A previous study claimed that MEL alleviated trinitrobenzene sulfonic acid-triggered colitis in rats via restraining the TLR4/NF-*κ*B pathway [37]. Yang et al. illustrated that MEL ameliorated intestinal damage, inflammation, and cognitive dysfunction induced by intestinal ischemia/reperfusion (I/R) through weakening the TLR4/Myd88 pathway [38]. Another research illuminated that MEL protected rats from small intestinal toxicity caused by radiotherapy through restraining the NF-*κ*B/NLRP3 pathway [39]. In this research, we discovered that MEL could decrease the TNF-*α* and IL-1*β* activities of the terminal ileum tissues in NEC mice. The further research demonstrated that the mRNA and protein levels of NLRP3, TLR4, NF-*κ*B, and caspase-1 of the terminal ileum tissues in NEC mice were largely intensified, whereas MEL blocked these changes. It was illuminated that MEL may blunt the level of intestinal tissue inflammation by repressing the expression of the NLRP3 inflammasome and TLR4 signaling, to further suppress the inflammatory factor production, thereby alleviating the inflammatory injury of intestinal tissue in NEC neonatal mice.

Karadag et al. discovered that increased inflammatory cytokines in NEC were also mainly caused by intestinal oxidative stress [40]. It was illuminated in another study that abnormal colonization of intestinal flora induced the increase in intestinal oxidative stress to damage the intestinal mucosal barrier, lead to bacterial translocation, and activate the immune system, which in turn triggered the NEC intestinal inflammation [29]. Premature infants with immature intestinal mucosal barrier function are prone to intestinal hypoxia, ischemia, abnormal colonization of flora, etc., and produce a large amount of oxygen free radicals to activate intestinal oxidative stress, resulting in protein, DNA, and cell membrane peroxidation [41]. Excessive free radicals can mediate the production of abundant MDA, resulting in tissue cell damage [42]. The first line of defense of the body's antioxidant system is responsible for inhibiting the generation of excess oxygen free radicals and lipid peroxidation. The defense of cells against oxidative stress is mediated by SOD and GSH [43]. SOD and GSH can neutralize oxygen free radicals to resist oxidative stress [44, 45]. Musa et al. pointed out that MEL evidently alleviated MDA level and enhanced SOD level of the irradiated intestinal tissues, thereby ameliorating radiotherapy-triggered intestinal injury [46]. One research by Cigsar et al. clarified that MDA activity was weakened, and SOD and GSH-Px levels were enhanced in NEC rats [47]; our results were consistent with Cigsar et al. Interestingly, MEL notably boosted the contents of SOD and GSH-Px and restrained the content of MDA in NEC mice.

## 5. Conclusion

In summary, this study illuminated that MEL could alleviate intestinal inflammatory response and protect intestinal injury of NEC by mitigating intestinal oxidative stress, increasing its antioxidant capacity, and weakening the NLRP3 inflammasome, which may offer a novel idea for the prevention and therapy of NEC.

## Data Availability

All data generated or analyzed during this study are included in this article.
